# Effectiveness of Daily Teriparatide in Managing Glucocorticoid-Induced Osteoporosis in Rheumatic Disease Patients After Switching From Bisphosphonate Therapy

**DOI:** 10.7759/cureus.82471

**Published:** 2025-04-17

**Authors:** Kaichi Kaneko, Makoto Kaburaki, Sei Muraoka, Kotaro Shikano, Nahoko Tanaka, Mai Kawazoe, Shinichi Kawai, Toshihiro Nanki

**Affiliations:** 1 Division of Rheumatology, Department of Internal Medicine, Toho University School of Medicine, Tokyo, JPN

**Keywords:** glucocorticoid, osteoporosis, osteoprotegerin, rankl, rheumatic diseases, teriparatide

## Abstract

Background: Osteoporosis is a serious complication of systemic glucocorticoid therapy. Bisphosphonates are commonly used to treat glucocorticoid-induced osteoporosis (GIOP) but may be ineffective. Teriparatide, a recombinant form of parathyroid hormone, stimulates bone formation and may be a promising alternative for patients who show an inadequate response to bisphosphonates. Nonetheless, evidence supporting the effectiveness of daily teriparatide in GIOP patients with an inadequate bisphosphonate response remains limited. Serum soluble receptor activator of nuclear factor-kappa B ligand (sRANKL) and osteoprotegerin (OPG) are important biomarkers of bone metabolism and may aid in understanding and treating various diseases. However, changes in serum sRANKL and OPG levels following the administration of teriparatide to GIOP patients remain unclear. Therefore, the present study investigated the effects of daily teriparatide on bone mineral density (BMD) and the biochemical markers of bone metabolism in rheumatic disease patients with GIOP.

Methods: This study included 23 patients with GIOP in rheumatic diseases. Patients were switched from oral bisphosphonates to daily teriparatide. Patients receiving a median daily dose of 5.0 mg of prednisolone were eligible for the present study. BMD at the lumbar spine was assessed before and six months after teriparatide therapy. Serum sRANKL, OPG, bone formation markers (bone alkaline phosphatase, osteocalcin, and procollagen type 1 N-terminal peptide), and bone resorption markers (crosslinked N-telopeptide of type I collagen and tartrate-resistant acid phosphatase isoform 5b) were measured during teriparatide therapy.

Results: Six months of treatment with teriparatide significantly increased lumbar spine BMD (before treatment 0.67 [0.60-0.74] and six months after treatment (0.71 {0.67-0.81} {median and interquartile range} g/cm2, p = 0.0337). Serum sRANKL levels significantly decreased after teriparatide therapy (0.07 {0.00-0.19} to 0.00 {0.00-0.07} pmol/L, p = 0.0182), while OPG levels remained unchanged (6.71 {5.79-8.13} to 7.18 {5.96-8.92} pmol/L, p = 0.588). The sRANKL/OPG ratio significantly decreased from baseline (0.62 {0.00-3.08}) to after teriparatide therapy (0.00 {0.00-0.11}, p = 0.0287). All serum bone formation markers and bone resorption markers increased after teriparatide therapy. No new vertebral fractures were detected.

Conclusions: Switching from bisphosphonates to daily teriparatide significantly increased lumbar spine BMD in rheumatic disease patients with GIOP. Sequential therapy with daily teriparatide after bisphosphonates may be an effective treatment for GIOP.

## Introduction

Glucocorticoids are useful for treating inflammatory and/or autoimmune diseases; however, their chronic use leads to adverse effects, including serious osteoporosis and related fractures. The American College of Rheumatology and the Japanese Society for Bone and Mineral Research recommend that patients treated with glucocorticoids at a dosage equivalent to prednisone or prednisolone 5 mg/day for at least three months receive bisphosphonates to inhibit osteoclast bone resorption [1,2]. Bisphosphonates have shown the most consistent efficacy in clinical trials on patients with glucocorticoid-induced osteoporosis (GIOP) [3]. However, since reduced bone formation is a key process in patients with GIOP, bisphosphonates may not be the ideal treatment for GIOP. Although bisphosphonates have been used to treat patients with GIOP, bone mineral density (BMD) does not improve in some patients [4].

Teriparatide, a recombinant form of parathyroid hormone, is a treatment option for GIOP, particularly in difficult-to-treat cases. Teriparatide is characterized by direct effects in bone formation through the stimulation of osteoblast activity and the inhibition of their apoptosis [5]. Teriparatide rapidly increases bone formation markers, and this is followed by elevations in bone resorption markers due to coupling effects [6,7]. We previously demonstrated that lumbar spine BMD was slightly higher and the incidence of new fractures was lower in GIOP patients who switched from bisphosphonates to weekly teriparatide than in those who continued bisphosphonates [8]. However, evidence for the effectiveness of daily teriparatide in GIOP patients with inadequate responses to bisphosphonates is insufficient.

Receptor activator for nuclear factor-κB ligand (RANKL) induces the differentiation and activation of osteoclasts to bind to receptor activator for nuclear factor-κB (RANK) expressed on osteoclast precursors or osteoclasts [9], and osteoprotegerin (OPG) is a decoy receptor for RANKL [10]. The RANKL/OPG ratio reflects the balance between bone resorption and inhibition. An elevated ratio indicates increased osteoclast activity, contributing to bone loss, whereas a reduced ratio suggests suppression of osteoclastic bone resorption [11]. Therefore, RANKL and OPG play an important role in regulating bone resorption by osteoclasts. We previously reported that serum soluble RANKL (sRANKL) levels may be a predictive factor for osteoporosis in patients with systemic autoimmune diseases receiving glucocorticoid therapy [12]. However, the effects of teriparatide on bone metabolism in GIOP, particularly on the RANKL-OPG system, remain unclear.

Therefore, the present study prospectively investigated patients with rheumatic diseases under glucocorticoid therapy who switched from bisphosphonates to daily teriparatide and aimed to clarify the efficacy of teriparatide on BMD and changes in sRANKL and OPG.

## Materials and methods

Methodology

Patients with rheumatic diseases who received daily teriparatide for the first time for GIOP between September 2012 and August 2015 at Toho University Omori Medical Center were enrolled. Patients with GIOP in rheumatic diseases who were switched from oral bisphosphonates to daily teriparatide were enrolled as the inclusion criteria. All patients were changed from oral bisphosphonates to daily subcutaneous injections of teriparatide (20 μg). GIOP was diagnosed based on guidelines released by the Japanese Society for Bone and Mineral Research for the management and treatment of GIOP in 2004 [2], which defined the following criteria for initiating treatment in patients 18 years of age and older: (1) presence of a prior fragility fracture; (2) BMD less than 80% of the young adult mean (YAM), even without prior fractures; (3) glucocorticoid therapy equivalent to ≥ 5 mg/day of prednisolone for ≥ 3 months, even in the presence of YAM is ≥ 80% and no prior fractures.

The inclusion criteria were as follows: (1) patients diagnosed with GIOP according to the Japanese Society for Bone and Mineral Research 2004 guidelines; (2) patients receiving glucocorticoid therapy equivalent to ≥5 mg/day of prednisolone for at least three months; (3) patients previously treated with oral bisphosphonates for at least six months but showing an inadequate response, defined as low BMD or the presence of fragility fractures; (4) patients who were subsequently switched to daily subcutaneous injections of teriparatide (20 μg/day). All patients met these criteria.

Participants were excluded from the study if they met any of the following criteria: (1) presence of metabolic bone diseases other than osteoporosis, including osteomalacia, Paget’s disease, or primary hyperparathyroidism; (2) a history of malignancy affecting bone metabolism; (3) severe renal impairment or end-stage renal disease requiring dialysis.

This was a prospective observational study that involved 23 patients with rheumatic diseases, including 10 with rheumatoid arthritis, seven with vasculitis syndrome, three with polymyalgia rheumatica, two with systemic lupus erythematosus, and one with dermatomyositis. The study protocol was approved by the Ethics Committee of Toho University Omori Medical Center (approval number: 24-97, dated September 13, 2022). All patients gave their written informed consent before enrollment.

Measurement of BMD

Before starting teriparatide therapy and after six months, the BMD of the lumbar spine (L2-4) was measured by dual-energy X-ray absorptiometry using Discovery A (Hologic, Waltham, MA, USA). BMD was automatically calculated from the bone area (cm^2^) and bone mineral content (g) and was expressed in g/cm^2^.

Serum biochemical markers

Serum levels of sRANKL (Biomedica, Vienna, Austria) and OPG (Biomedica) were measured by enzyme-linked immunosorbent assays (ELISA). As bone formation markers, serum levels of bone alkaline phosphatase (BAP; Quidel, San Diego, CA, USA) were measured by an enzyme immunoassay, and osteocalcin (OC; Mitsubishi Kagaku Bio-Clinical Laboratories, Tokyo, Japan) and procollagen type 1 N-terminal peptide (P1NP; Orion Diagnostica, Espoo, Finland) by immunoradiometric assays. As bone resorption markers, serum levels of the crosslinked N-telopeptide of type I collagen (NTX; Inverness, Princeton, NJ, USA) and tartrate-resistant acid phosphatase isoform 5b (TRACP-5b; DS Pharma Biomedical, Tokyo, Japan) were measured by ELISA. Fasting morning blood samples were collected from patients before the initiation of treatment and after six months of teriparatide therapy.

Statistical analysis

Statistical analyses were performed with Prism, version 9.0 software (Graphpad Software, San Diego, CA). Numerical data were expressed as medians with interquartile ranges. When comparing the two groups, the Mann-Whitney U test was applied for numerical data. Multiple comparisons were performed by the Kruskal-Wallis test. In all analyses, p <0.05 was considered to be significant.

## Results

Patient characteristics

Table 1 shows the demographic and clinical data of patients. The median age of patients was 69.0 years. The median BMD at the lumbar spine of patients was 0.67 g/cm2, and the median T-score was −3.10 at baseline. Despite following bisphosphonate use, all patients had a high risk of bone fragile fractures, as defined by a T-score of −2.5 standard deviations (SD) or less or ≥2 prevalent fragility fractures independently of BMD values [13,14]. Rheumatoid arthritis was the underlying disease in 10 of the enrolled patients (43.5%). All patients had been treated with prednisolone (2-20 mg/day {median: 5.0 mg/day}) at the initiation of teriparatide therapy. The median duration of prednisolone therapy prior to the start of teriparatide was 48 months (range: 12-156 months).

**Table 1:** Demographics and clinical data of the study population at baseline. Data are expressed as the median with 25th to 75th percentiles. CRP: C-reactive protein; BAP: bone alkaline phosphatase; OC: osteocalcin; P1NP: procollagen type 1 N-terminal peptide; NTX: crosslinked N-telopeptide of type I collagen; TRACP-5b: tartrate-resistant acid phosphatase isoform 5b.

	Patients (n = 23)
Age (years)	69.0 (65.0–76.0)
Number of males/females	1/22
Postmenopausal (%)	22 (100)
Body mass index (kg/m^2^)	20.3 (17.8–24.1)
Prednisolone dose (mg/day)	5.0 (2.0–10.0)
Duration of glucocorticoids (months)	72.0 (28.0–108.0)
Duration of bisphosphonate treatment (months)	54.8 (21.8–75.6)
Frequency of prior fragility fractures (%)	23 (100)
No. with 2 or more prevalent fragility fractures (%)	11 (47.8)
Lumbar spine bone mineral density (g/cm^2^)	0.67 (0.60–0.74)
T-score at the lumbar spine	−3.10 (−3.75–−2.55)
CRP (mg/dL)	0.1 (0.1–1.0)
BAP (U/L)	10.9 (8.8–16.0)
OC (ng/mL)	3.6 (2.9–5.8)
P1NP (μg/L)	26.8 (12.5–43.2)
NTX (nmol BCE/L)	12.7 (10.7–22.4)
TRACP-5b (mU/dL)	227.5 (162.5–505.5)
Rheumatic diseases	
-Rheumatoid arthritis	10 (43.5%)
-Vasculitis syndrome	7 (30.4%)
-Polymyalgia rheumatica	3 (13.0%)
-Systemic lupus erythematosus	2 (8.7%)
-Dermatomyositis	1 (4.4%)

BMD

**Figure 1:**
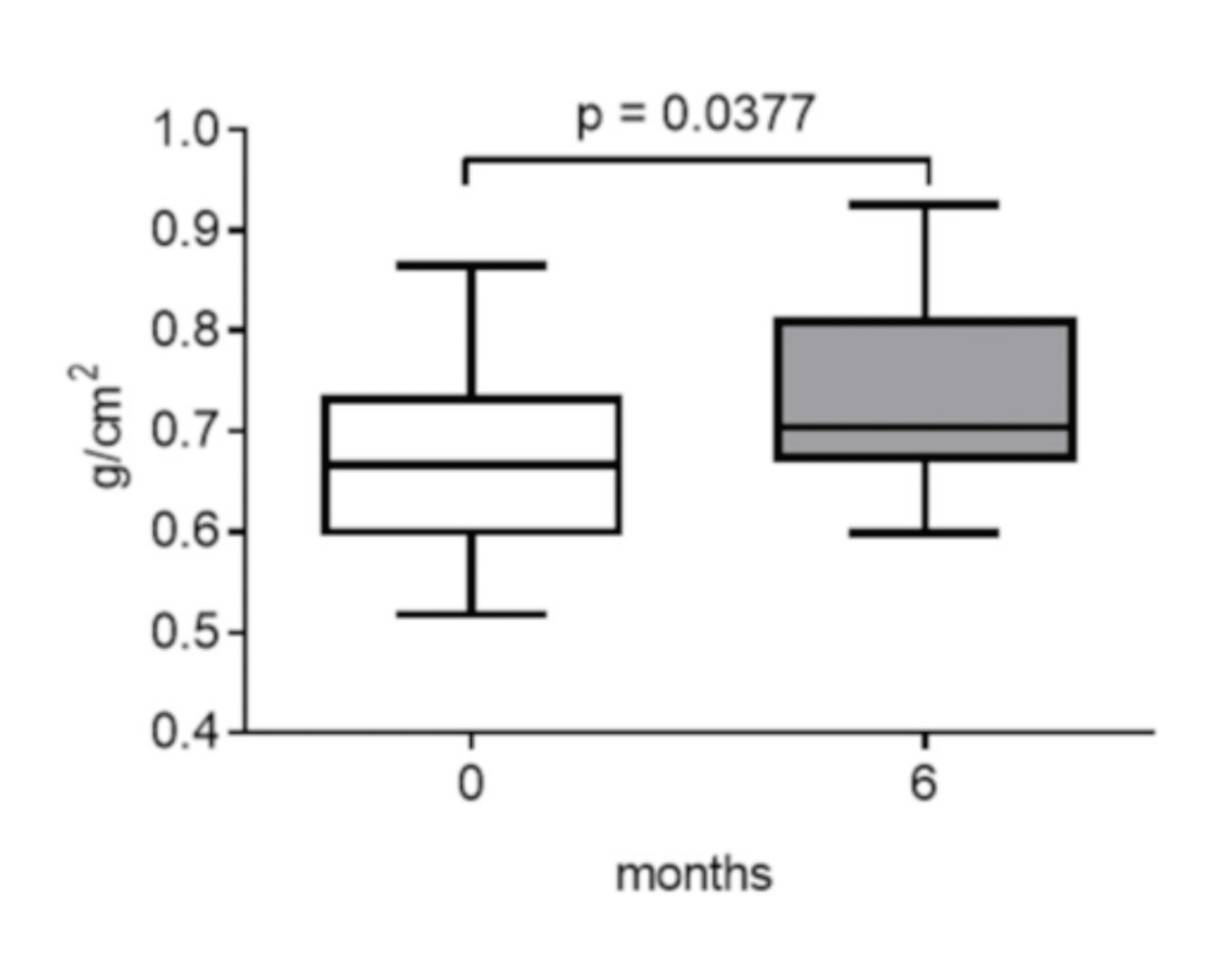
Changes in lumbar spine BMD six months after teriparatide therapy. Data are expressed as the median with 25th to 75th percentiles. BMD: bone mineral density

Treatment with teriparatide significantly increased the median BMD of patients at six months (0.67 [0.60-0.74] to 0.71 [0.67-0.81] g/cm^2^) (p = 0.0377) (Figure 1).

**Figure 2:**
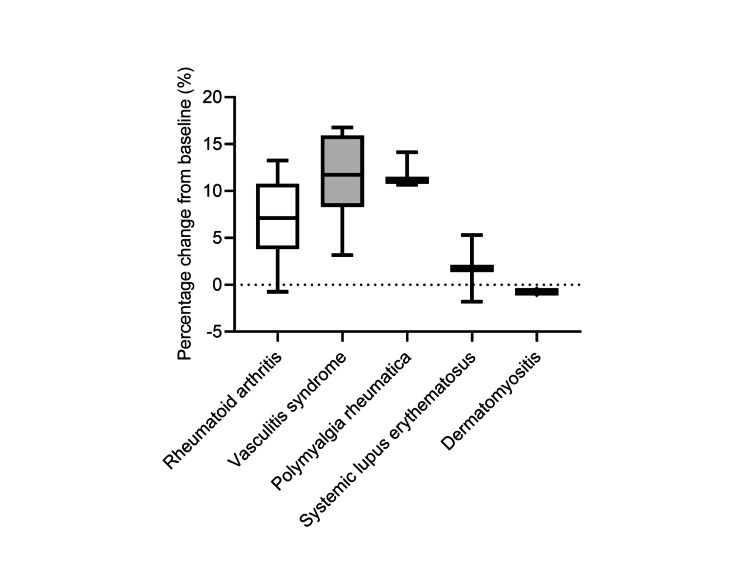
Changes in lumbar spine BMD six months after teriparatide therapy in patients with different rheumatic diseases Data are expressed as the median with 25th to 75th percentiles. BMD: bone mineral density

The changes of BMD among rheumatic diseases appear to exhibit some differences; however, these differences are not statistically significant (Figure 2).

Serum sRANKL and OPG

**Figure 3:**
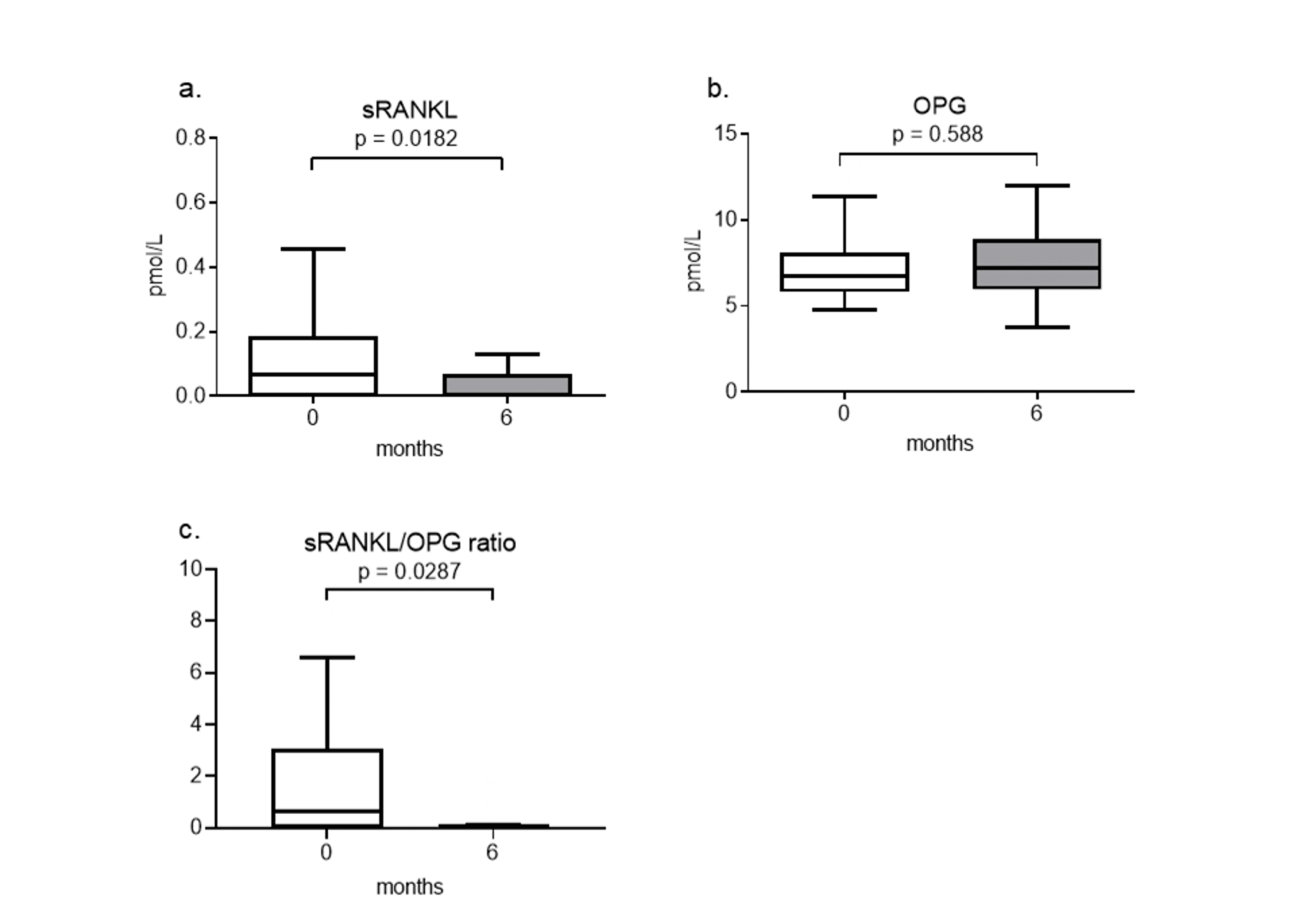
Changes in serum sRANKL (a) and OPG (b) levels and the sRANKL/OPG ratio (c) six months after teriparatide therapy Data are expressed as the median with 25th to 75th percentiles. sRANKL: soluble receptor activator for nuclear factor-κB ligand, OPG: osteoprotegerin

Serum sRANKL levels significantly decreased from baseline (0.07 {0.00-0.19} pmol/L) to six months after teriparatide therapy (0.00 {0.00-0.07} pmol/L) (p = 0.0182) (Figure 3a). In contrast, serum OPG levels did not significantly change from baseline (6.71 {5.79-8.13} pmol/L) to after teriparatide therapy (7.18 {5.96-8.92} pmol/L) (p = 0.588) (Figure 3b). The RANKL/OPG ratio significantly decreased from baseline (0.62 {0.00-3.08}) to after teriparatide therapy (0.00 {0.00-0.11}) (p = 0.0287) (Figure 3c).

Serum bone turnover markers

**Figure 4:**
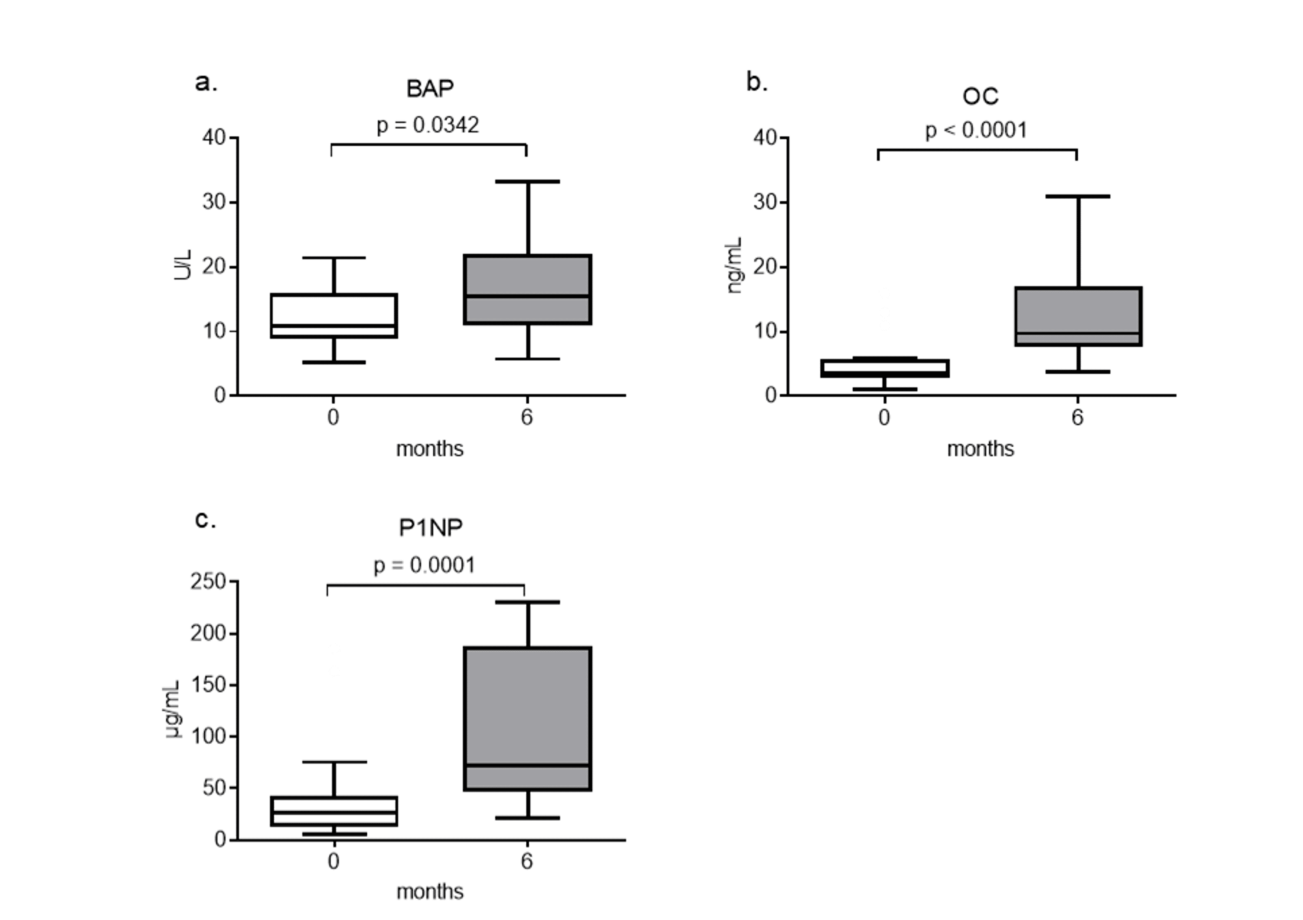
Changes in serum bone formation markers after teriparatide therapy The serum levels of BAP (a), OC (b), and P1NP (c) are shown. Data are expressed as the median with 25th to 75th percentiles. BAP: bone alkaline phosphatase, OC: osteocalcin, P1NP: procollagen type 1 N-terminal peptide

Serum levels of the bone formation markers, BAP, OC, and P1NP, significantly increased after six months of teriparatide therapy (Figures 4a-4c).

**Figure 5:**
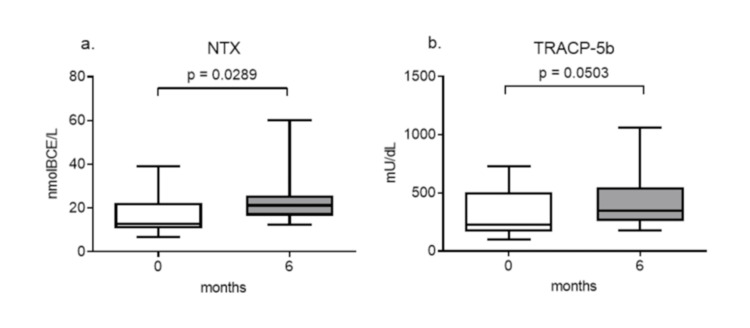
Changes in serum bone resorption markers after teriparatide therapy The serum levels of NTX (a) and TRACP-5b (b) are shown. Data are expressed as the median with 25th to 75th percentiles. NTX: crosslinked N-telopeptide of type I collagen, TRACP-5b: tartrate-resistant acid phosphatase isoform 5b

Serum levels of the bone resorption markers NTX and TRACP5b also increased after the treatment; however, the increase in TRACP5b was not significant (Figures 5a, 5b).

Adverse events

No new vertebral fractures were detected by radiography during the six months of teriparatide treatment. No cases of hypercalcemia, nausea, or other serious adverse events were observed during the observed period.

## Discussion

The present study showed that a switch from bisphosphonates to the daily teriparatide treatment significantly increased lumbar spine BMD in rheumatic disease patients with GIOP. We found that serum sRANKL levels significantly decreased, while serum OPG levels remained unchanged after the teriparatide treatment. Daily teriparatide increased bone formation and resorption marker levels, suggesting improvements in bone remodeling.

A meta-analysis of randomized controlled trials recently showed the superior efficacy of daily teriparatide over bisphosphonates for GIOP but examined drug-naive patients with osteoporosis [15]. Hirooka et al. reported that 24 months of daily teriparatide increased BMD at the lumbar spine and femoral neck, whereas denosumab increased BMD only at the lumbar spine in rheumatic disease patients with GIOP and following bisphosphonate treatment [16]. In the present study, daily teriparatide significantly increased lumbar spine BMD at six months in patients with rheumatic diseases following treatment with bisphosphonates but with GIOP.

RANKL and OPG regulate osteoclast formation and activation [17]. A previous study reported that one month of daily treatment with teriparatide significantly increased serum sRANKL, whereas serum OPG remained unchanged in patients with postmenopausal osteoporosis [18]. In the present study, we showed that treatment with teriparatide decreased serum sRANKL levels and the sRANKL/OPG ratio in GIOP patients. Glucocorticoids may induce different changes in sRANKL levels. Teriparatide-induced reductions in sRANKL may contribute to increases in BMD.

Teriparatide stimulates both osteoclast-mediated bone resorption and osteoblast-mediated bone formation. This increased bone turnover is evidenced by marked increases in the biochemical markers of both bone formation and resorption beginning soon after the initiation of teriparatide treatment for GIOP [19-21]. The increases observed in BAP, OC, and P1NP, bone formation markers, and TRAP-5b and NTX, bone resorption markers, with the daily teriparatide treatment in rheumatic patients with GIOP in the present study were consistent with previous findings.

Recent studies have explored the comparative efficacy of other treatment options for osteoporosis, including romosozumab and denosumab. A study in postmenopausal osteoporosis showed that romosozumab achieved greater increases in BMD following bisphosphonate therapy compared to denosumab or teriparatide [22]. The finding indicated that romosozumab led to the most significant BMD improvement, followed by teriparatide and denosumab. Additionally, we recently conducted a randomized controlled study comparing the effects of romosozumab, denosumab, and bisphosphonates in patients with rheumatic diseases who were newly initiating glucocorticoid therapy [23]. The results demonstrated that romosozumab produced the most significant increase in lumbar spine BMD at 12 months, suggesting that targeting the Wnt/β-catenin signaling pathway through sclerostin inhibition may be an effective approach for GIOP. These findings support the potential use of romosozumab in high-risk osteoporosis patients, including those previously treated with bisphosphonates. We acknowledge the significance of comparing denosumab, romosozumab, and teriparatide in GIOP and plan to investigate this in future studies.

However, there are several limitations that need to be addressed. Since the present study was conducted at a single hospital and the sample size was small, it may not accurately represent the broader population from which it was drawn. Furthermore, the lack of a control group in the present study limits the direct evaluation of the efficacy and safety of the treatment and may be subject to bias and confounding factors. Although teriparatide is typically prescribed for a duration of up to 24 months in Japan, we selected a six-month observation period to evaluate its early effects on BMD and bone metabolism in patients with GIOP. This timeframe was informed by a previous study that demonstrated significant changes in lumbar spine BMD and bone turnover markers within the first six months of treatment [20]. However, the follow-up period was limited to six months, which restricts our ability to assess the long-term efficacy of teriparatide in increasing BMD. The current small sample size and short-term observation limit the ability to analyze the effects of preventing new fractures. We did not assess femoral neck BMD for the following reasons: First, the effects of teriparatide are more pronounced in trabecular-rich regions, such as the lumbar spine, making it the preferred site for short-term evaluation. Second, some patients had pre-existing hip joint pathology or had undergone total hip arthroplasty, which hindered accurate and consistent measurement of femoral BMD in this population. Consequently, we were unable to evaluate the effect of teriparatide in the femoral BMD. A larger sample size, along with multicenter, long-term observations, including femoral BMD analysis and a control treatment, will be necessary in the future. This will enable a multivariate analysis to examine the factors that influence the increase in BMD with teriparatide in patients with rheumatic diseases.

## Conclusions

This study demonstrated that switching from bisphosphonates to daily teriparatide significantly increased lumbar spine BMD in patients with rheumatic diseases with GIOP. Furthermore, the daily teriparatide treatment significantly decreased serum sRANKL levels without changing serum OPG levels, resulting in a reduced RANKL/OPG ratio. These results suggest the potential benefits of sequential therapy using daily teriparatide following bisphosphonates treatment for GIOP.
